# Clinical worsening in adult congenital heart disease and heart failure: A single-centre, observational study^☆^

**DOI:** 10.1016/j.ijcchd.2026.100656

**Published:** 2026-01-27

**Authors:** Thibault Bourgeois, Juliette Hubert, Pieter De Meester, Thibault Petit, Els Troost, Lucas Van Aelst, Filip Rega, Philip Moons, Werner Budts, Alexander Van De Bruaene

**Affiliations:** a Division of Cardiology, University Hospitals Leuven, Leuven, Belgium; b Faculty of Medicine, UCLouvain, Brussels, Belgium; c Division of Congenital and Structural Cardiology, University Hospitals Leuven, Leuven, Belgium; d Division of Cardiac Surgery, University Hospitals Leuven, Leuven, Belgium; e Department of Public Health and Primary Care, KU Leuven, Leuven, Belgium; f University of Gothenburg Centre for Person-Centred Care (GPCC), Sahlgrenska Academy, University of Gothenburg, Gothenburg, Sweden; g Department of Paediatrics and Child Health, University of Cape Town, Cape Town, South Africa

**Keywords:** ACHD-HF, Prognosis and outcome, Medical therapy

## Abstract

**Background:**

Data on contemporary treatment approaches and reliable markers of clinical worsening in adult patients with congenital heart disease and heart failure (ACHD-HF) are scarce. This study aimed at evaluating (1) medical therapy within a contemporary ACHD-HF cohort and (2) the incidence of various (composite) endpoints across anatomical and pathophysiological subgroups.

**Methods:**

Retrospective study including ACHD-HF patients (≥16 years) under active follow-up at a tertiary care center, monitored until last visit, death, ventricular assist device (VAD) implantation, or heart transplant (HTX). Medical therapy was documented at time of inclusion and final follow-up. Clinical endpoints were evaluated, after excluding patients with early events (<1 week of inclusion) or without follow-up. Endpoints included HF hospitalization, increase of diuretic treatment, NYHA class progression, all-cause mortality, HTX and VAD placement.

**Results:**

Of the 256 ACHD-HF patients (mean age 50 ± 17 years; 52 % male), 56 were excluded for the final analysis. Two hundred patients were followed for a median of 30 (IQR 22–36) months. Changes in medical therapy included: increased use of mineralocorticoid receptor antagonists (p = 0.038), sodium-glucose transport protein 2 inhibitors (p = 0.002) and a trend towards increased use of angiotensin receptor-neprilysin inhibitors (p = 0.070). Furthermore, whereas HF hospitalization (p < 0.001) and progression of NYHA (p = 0.016) were associated with death, whereas HTX or VAD implant and escalation of diuretic therapy (p = 0.961) were not.

**Conclusions:**

This study characterizes current ACHD-HF therapy and identifies NYHA progression and HF hospitalization as potential markers of clinical worsening as they relate to death, HTX, and VAD implantation.

## Highlights

• The incidence of adverse clinical outcomes across distinct anatomical and pathophysiological subgroups of adult patients with congenital heart disease and heart failureremains high.
• Both worsening of NYHA functional class, and HF hospitalization were significantly associated with an increased risk of the composite endpoint comprising all-cause mortality, HTX, and/or VAD implant and appear valuable endpoints to assess interventions in ACHD-HF patients.
• In contrast, escalation of diuretic therapy did not demonstrate a statistically significant association with adverse outcomes, suggesting limited value as a surrogate endpoint in clinical trials.

## 1 Introduction

Advancements in medical management and surgical techniques have significantly improved the survival of children born with congenital heart defects. As a result, congenital heart disease (CHD) has evolved from being primarily a pediatric condition to one with substantial adult representation [1,2]. In Belgium, estimates indicate that approximately 90 % of children with CHD reach adulthood [3], which is in line with trends worldwide [4]. With improved survival rates and despite advancements in surgical techniques, HF has emerged as a major concern in adults with CHD. Observational studies report an overall prevalence of adult congenital heart disease patients with heart failure (ACHD-HF) of 6.4 %, along with a significantly increased short-term all-cause mortality compared to matched controls [5,6].

Management strategies for ACHD-HF emphasize structural and electrical optimization prior to

standard pharmacological therapy [7]. Although hindered by small sample sizes and the marked anatomic and physiological heterogeneity, a number of small studies have reported favourable outcomes with angiotensin receptor-neprilysin inhibitors (ARNI) and sodium-glucose cotransporter-2 inhibitors (SGLT2i) in selected ACHD-HF cohorts [8,9].

Nevertheless, real-world evidence on practice patterns for the management of ACHD-HF remains scarce. Furthermore, for the design of clinical trials evaluating the efficacy of interventions, information on the incidence and significance of different (combinations of) clinical endpoints would be of interest.

Therefore, this study aimed to evaluate [1] changes in medical therapy within a contemporary cohort of ACHD-HF patients, and [2] the incidence of various (composite) endpoints across distinct anatomical and pathophysiological subgroups, and to explore whether ‘softer’ or surrogate endpoints could serve as valid parameters of clinical worsening and potentially replace/complement traditional ‘hard’ endpoints.

## 2 Methods

### 2.1 Study population

We conducted an observational study with a retrospective design, which included 256 ACHD-HF patients (≥16 years) under active follow-up at a tertiary care center (University Hospitals Leuven, Leuven, Belgium). First contact and inclusion was determined by a single investigator (A. V. D. B.). Patients were followed until latest known follow-up visit or until death, ventricular assist device (VAD) placement and/or heart transplant (HTX). Demographic and clinical data were retrieved from our electronic patient management system. Medical therapy was collected during first and final follow-up. Patients were stratified into mutually exclusive subgroups based on the classification system proposed by Task Force 1 of the 32nd Bethesda Conference [6,10]. The subgroups were defined as follows: cyanotic ACHD (including those with Eisenmenger physiology) (subgroup 1); Fontan circulation (subgroup 2); biventricular circulation with a systemic right ventricle (RV) (subgroup 3); biventricular circulation with a systemic left ventricle (LV) further classified into three subgroups; those with shunt lesions (subgroup 4); those with predominant right-sided residual lesions (subgroup 5); and those with predominant left-sided residual lesions (subgroup 6).

### 2.2 Adult congenital heart disease-heart failure definition

ACHD-HF was defined according to previously published criteria [6,11] as the presence of signs and/or symptoms of HF necessitating medical treatment, in combination with at least one of the following: evidence of ventricular dysfunction (systolic and/or diastolic) accompanied by elevated intracardiac filling pressures (I); elevated levels of B-type natriuretic peptide (BNP) or N-terminal proBNP (II); peak oxygen consumption within the lowest quartile based on normative data for specific ACHD subtype (III); or the presence of one or more of four distinctive manifestations in patients with a Fontan circulation (protein loosing enteropathy, plastic bronchitis, a Fontan systemic venous pressure ≥20 mmHg, or a cardiac index <2 L/min/m^2^) (IV). All HF diagnoses were assessed by a single investigator (A. V. D. B.) at the time of patient inclusion. In cases of diagnostic uncertainty, the classification was reviewed in consultation with co-investigators (W. B., E. T., and P. D. M.).

### 2.3 Clinical endpoints definition

Endpoints assessed included HF hospitalization, diuretics escalation and/or NYHA progression in addition to all-cause mortality, HTX and VAD placement. Hospitalization due to HF was defined by clinical symptoms/signs of congestion requiring (temporary) intensification of (intravenous) diuretics until adequate decongestion was achieved. Escalation of diuretic therapy in the outpatient setting was defined as a sustained increase of loop diuretics dosage and/or association of different class of diuretics (thiazide diuretics, mineralocorticoid receptor antagonists (MRA) or carbonic anhydrase inhibitors) at last clinical contact compared with the contact at inclusion. NYHA progression was defined as a persistent increase in NYHA functional class (or dyspnea symptoms) during the different follow-up visits. Data collection was conducted in accordance with the General Data Protection Regulation (GDPR). The study protocol received approval from the institutional ethics committee and was carried out in compliance with the ethical principles outlined in the Declaration of Helsinki.

### 2.4 Statistical analysis

Statistical analyses were conducted using SPSS ® for Windows (version 29, IBM Corp., USA). Descriptive data for continuous variables are reported as mean ± standard deviation (SD) or as median with interquartile range (IQR), depending on data distribution. Discrete variables are presented as absolute frequencies and corresponding percentages. McNemar's test was used to test matched pairs. Statistical outcome analysis was performed after exclusion of patients with an early event (<1 week of inclusion) or insufficient follow-up (e.g. no follow-up contacts after inclusion). Kaplan-Meier survival curves were generated to evaluate time-to-event outcomes for relevant clinical endpoints and differences in event-free survival between groups were assessed via the log-rank test.

## 3 Results

### 3.1 Patient characteristics and medical therapy

**Fig. 1:**
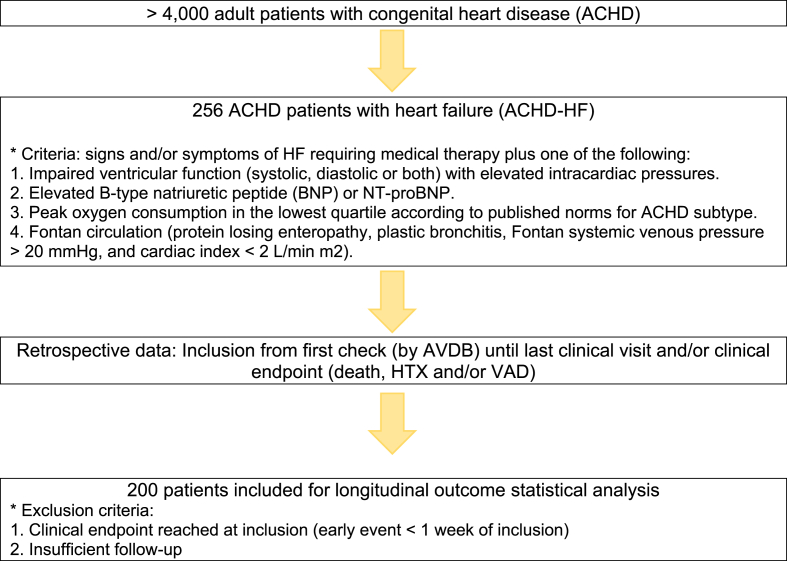
Summarized overview regarding the inclusion of ACHD patients. HF: heart failure – ACHD: adults with congenital heart disease – HTX: heart transplantation – VAD: ventricular assist device.

A total of 256 patients (mean age 50 ± 17 years) were diagnosed with ACHD-HF, from a larger cohort of over 4000 individuals with ACHD followed at a tertiary academic medical center. Fifty-six patients were excluded as they had either an early event (<1 week of inclusion) or insufficient follow-up (e.g. no follow-up contacts after inclusion). Of the 200 remaining patients, 54 % were male. According to the Bethesda classification around 17 % of the patients were categorized as having simple defects, while approximately 43 % had lesions of moderate complexity and 40 % had lesions of severe complexity. Detailed baseline characteristics of the ACHD-HF cohort are presented in Table 1Fig. 1

**Table 1:** Baseline characteristics of the entire ACHD-HF cohort. *Abbreviations:* ACHD HF, adult congenital heart disease with heart failure; FU, follow-up; SD, standard deviation; IQR, interquartile range; BMI, body mass index; SBD, systolic blood pressure; DBP, diastolic blood pressure; bpm, beats per minute; 1V, univentricular heart; 2V-RV, biventricular heart with systemic right ventricle; 2V-LV, biventricular heart with systemic left ventricle; VACTERL, vertebral defects, anal atresia, cardiac defects, tracheoesophageal fistula, renal anomalies, and limb abnormalities; CHARGE, coloboma, heart defects, atresia choanae, retardation of growth and/or development, genital hypoplasia, and ear anomalies; RF, radiofrequency; CRT, cardiac resynchronization therapy; AICD, automatic implantable cardioverter-defibrillator; ACEi, angiotensin-converting enzyme inhibitor; ARB, angiotensin receptor blocker; ARNi, angiotensin receptor-neprilysin inhibitor; MRA, mineralocorticoid receptor antagonist; SGLT2i, sodium-glucose cotransporter 2 inhibitor; CA inhibitor, carbonic anhydrase inhibitor; HTX, heart transplantation; VAD, ventricular assist device.∗excluding those with early events.

Variable	ACHD HF n = 256	ACHD HF∗ n = 200
Male gender n (%)	132 (51.6)	108 (54.0)
Mean age at last follow up (years)	49.5 (SD 16.7)	48.7 (SD 15.6)
Median follow up time (months)	27.8 (IQR 5–34)	30.3 (IQR 21.6–36.0)
Mean BMI (kg/m2)	25.4 (SD 5.6) (n = 231)	25.8 (SD 5.5) (n = 184)
Median SBP (mmHg)	119 (IQR 107–133) (n = 242)	119 (IQR 106–133) (n = 194)
Median DBP (mmHg)	68 (IQR 60–78) (n = 242)	68 (IQR 60–76) (n = 194)
Median Heart rate (bpm)	72 (IQR 64–82) (n = 242)	72 (IQR 63–81) (n = 191)
Bethesda classification		
Mild n (%)	43 (16.7)	34 (17.0)
Moderate n (%)	116 (45.3)	86 (43.0)
Severe n (%)	97 (38.0)	80 (40.0)
Classification based on circulation		
Cyanotic/Eisenmenger	44 (17.2)	31 (15.5)
1 V	31 (12.1)	26 (13.0)
2V-RV	31 (12.1)	25 (12.5)
2V-LV	150 (58.6)	118 (59.0)
Other classification (subgroups)		
Cyanotic/Eisenmenger	44 (17.2)	31 (15.5)
Fontan	31 (12.1)	26 (13.0)
Systemic RV	31 (12.1)	25 (12.5)
Shunt lesions	49 (19.1)	38 (19.0)
Predominant right sided	75 (29.3)	59 (29.5)
Predominant left sided	26 (10.2)	21 (10.5)
Intervention n (%)	213 (83.2)	168 (84)
Any genetic abnormality n (%)	25 (9.8)	21 (10.5)
Down n (%)	13 (5.1)	10 (5.0)
Noonan n (%)	1 (0.4)	1 (0.5)
22q11 n (%)	6 (2.3)	6 (3.0)
Williams n (%)	1 (0.4)	0 (0)
Holt Oram n (%)	2 (0.8)	2 (1.0)
VACTERL n (%)	1 (0.4)	1 (0.5)
CHARGE n (%)	1 (0.4)	1 (0.5)
Coronary artery disease n (%)	7 (2.7)	7 (3.5)
Atrial arrhythmia n (%)	133 (52.0)	96 (48.0)
RF ablation n (%)	55 (21.5)	45 (22.5)
Ventricular arrhythmia n (%)	36 (14.1)	31 (15.5)
Pacemaker n (%)	83 (32.4)	66 (33.0)
CRT n (%)	15 (5.9)	10 (5)
AICD n (%) (1 missing)	33 (12.9)	30 (15.1)
Medication at inclusion		
ACEi n (%)	81 (31.7)	66 (33.0)
ARB n (%)	26 (10.2)	22 (11.0)
ARNI n (%)	7 (2.7)	6 (3.0)
Beta blocker n (%)	173 (67.6)	137 (68.5)
MRA n (%)	147 (57.4)	111 (55.5)
SGLT2i n (%)	3 (1.2)	3 (1.5)
Loop diuretic n (%)	187 (73.1)	140 (70.0)
Thiazide diuretic n (%)	27 (10.5)	23 (11.5)
CA inhibitor n (%)	5 (2.0)	2 (3.0)
Medication at last FU		
ACEi n (%)	74 (28.9)	59 (29.5)
ARB n (%)	25 (9.8)	21 (10.5)
ARNI n (%)	13 (5.1)	12 (6.0)
Beta blocker n (%)	170 (66.4)	133 (66.5)
MRA n (%)	159 (62.1)	123 (61.5)
SGLT2i n (%)	15 (5.9)	15 (7.5)
Loop diuretic n (%)	189 (73.8)	142 (71)
Thiazide diuretic n (%)	29 (11.3)	25 (12.5)
CA inhibitor n (%)	5 (2.0)	3 (1.5)
Clinical endpoint		
Death n (%)	81 (31.6)	40 (20.0)
HTX n (%)	18 (7.0)	10 (5.0)
VAD n (%)	5 (1.9)	5 (2.5)
Death, HTX and/or VAD n (%)	100 (39.1)	51 (25.5)

**Fig. 2:**
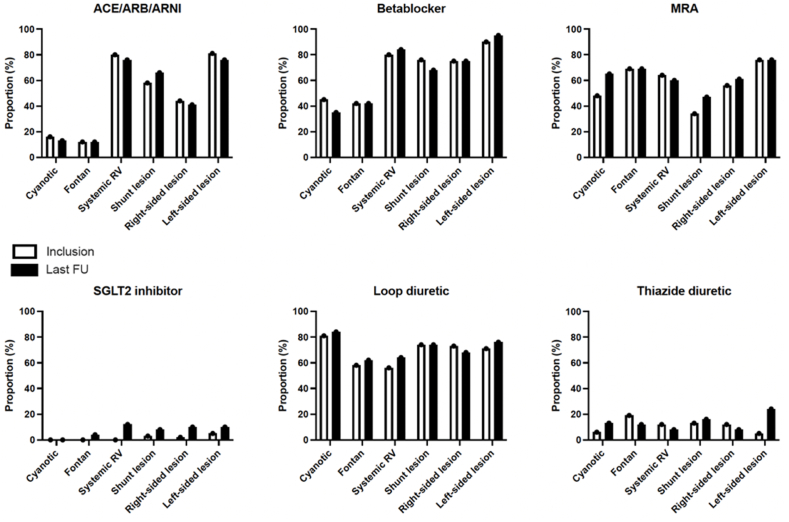
A graphical representation of the medical data stratified by ACHD subtype. Therapeutic (medical) adjustments over time revealed a statistically significant increase in the use of mineralocorticoid receptor antagonists (MRA) (p = 0.038) and sodium-glucose transport protein 2 inhibitors (SGLT2i (p = 0.002), along with a trend towards increased prescription of angiotensin receptor-neprilysin inhibitors (ARNI) (p = 0.070) for the entire cohort. There was a trend towards increased prescription of MRA in patients with shunt lesions (p = 0.063) and SGLT2i in patients with residual right-sided lesions (p = 0.063).

At the last follow-up, of the 200 patients included in the longitudinal follow-up analysis, almost 40 % of patients received an angiotensin-converting enzyme inhibitor (ACEi), angiotensin receptor blocker (ARB) or ARNI; around 67 % of patients received beta-blockers (BB); 62 % were on MRA and 71 % were on loop diuretics. Changes in medical therapy are presented in Fig. 2. Overall, there was a statistically significant increase in the use of MRA (p = 0.038) and SGLT2i (p = 0.002), along with a trend towards increased prescription of ARNI (p = 0.070).

### 3.2 Outcome

**Fig. 3:**
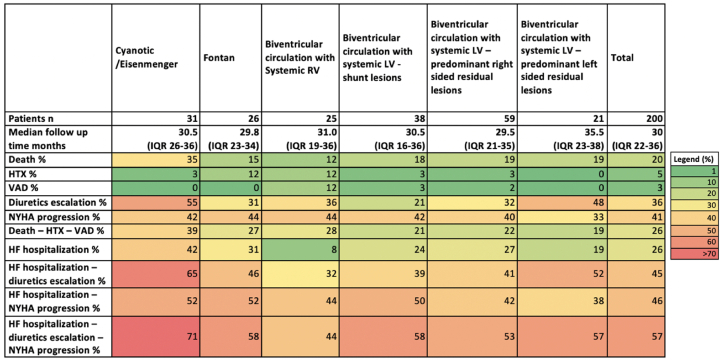
A heatmap depicts the incidence of various clinical endpoints – both individual and composite - across predefined anatomical and pathophysiological subgroups. HTX: heart transplantation – VAD: ventricular assist device – HF: heart failure – NYHA: New York Heart association – LV: left ventricle – RV: right ventricle.

A total of 200 patients were included in the longitudinal follow-up analysis, with a median follow-up of 30 months (IQR 22–36 months). Fig. 3 depicts the incidence of individual and composite endpoints across predefined anatomical and pathophysiological subgroups.

Patients with cyanotic congenital heart disease had the highest adverse outcomes event rates, with a mortality rate of 14 % per year, a HF-hospitalization rate of 16.8 % per year and a composite endpoint HF-hospitalization, diuretics escalation and NYHA progression of 28.4 % per year. Overall, event rates for the combined endpoint (all-cause mortality, HTX or VAD implant) as well as for the occurrence of a HF hospitalization were 10 % per year.

Interestingly, patients with a systemic right ventricle had a lower all-cause mortality, but a higher proportion of advanced HF interventions (HTX and VAD implantation). Furthermore, this subgroup had the lowest incidence of HF hospitalizations (8 %), while all other subgroups experienced hospitalization rates of 19 % or higher over the follow-up duration.

**Fig. 4:**
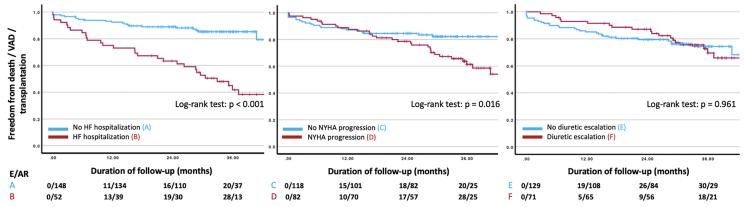
Kaplan-Meier survival analysis demonstrated significant differences in event-free survival (freedom from death, heart transplantation, or VAD implantation) according to heart failure hospitalization (p < 0.001) and NYHA functional class progression (p = 0.016). Notably, escalation of diuretic therapy was not associated with a difference in survival (p = 0.961). VAD: ventricular assist device - E: endpoint – AR: at risk.

Survival analysis, as illustrated in Kaplan-Meier survival curves (Fig. 4), demonstrated significant differences in event-free survival (freedom from death, heart transplantation or VAD implantation) according to heart failure hospitalization (p < 0.001) and NYHA functional class progression (p = 0.016). Notably, escalation of diuretic therapy was not associated with a difference in survival (p = 0.961).

## 4 Discussion

This study provides novel insights into contemporary HF management among adults with CHD, highlighting current pharmacological practice patterns as well as the incidence of adverse clinical outcomes across distinct anatomical and pathophysiological subgroups. Notably, both worsening of NYHA functional class, and HF hospitalization were significantly associated with an increased risk of the composite endpoint comprising all-cause mortality, HTX, and/or VAD implant and appear valuable endpoints to assess interventions in ACHD-HF patients. In contrast, escalation of diuretic therapy did not demonstrate a statistically significant association with adverse outcomes, suggesting limited value as a surrogate endpoint in clinical trials.

### 4.1 Medical therapy in ACHD-HF

Unlike acquired HF, where evidence-based guidelines are primarily supported by large randomized controlled trials [12,13], data guiding optimal medical therapy in ACHD-HF patients remain limited, largely due to the absence of trials evaluating hard clinical endpoints. Current consensus suggests that, in ACHD-HF patients with a biventricular circulation and reduced systemic ventricular ejection fraction, guideline-directed medical therapy (e.g. BB, MRA, ACEi/ARB/ARNI, digoxin, diuretics) may be applied (i.e. patients with left-sided lesions and symptomatic patients with a systemic right ventricle) [14,15]. Indeed, although evidence regarding the effect of ACEi, ARB, BB and MRA in adults with a systemic RV is conflicting, recent studies tend to support the use of guideline-directed HF therapy in these patients with positive effects on functionality (NYHA), NT-proBNP levels and ventricular function [[16], [17], [18], [19], [20], [21], [22]]. In contrast, evidence supporting medical therapy in ACHD-HF patients with preserved ejection fraction, cyanotic CHD or single ventricular disease is very limited, aside from the symptomatic use of diuretics [14,23] and emerging data on the use of SGLT2i in ACHD patients in general and in Fontan circulation more specifically [24,25].

Consistent with these data, our entire cohort demonstrated a small, but significant increase in the use of MRA (p = 0.038), SGLT2i (p = 0.002) and a trend toward greater prescription of ARNIs (p = 0.070) over the study period. BBs were administered in about 65 % of patients, potentially reflecting a high burden of atrial (52 %) and ventricular (14 %) arrhythmia [26]. Guideline-directed HF therapy was most prevalent in ACHD-HF patients with a systemic RV and those with a biventricular circulation and left-sided lesions. Although use was increasing, adoption of SGLT2i was still limited, probably related to reimbursement of these drugs in a clinical setting. Patients with a Fontan circulation were mainly treated with MRA and diuretics.

This study reflects the evolving therapeutic landscape in ACHD-HF management and provides real-world evidence of the (increasing) incorporation of guideline-directed HF therapy in some subgroups of ACHD patients, albeit supported primarily by observational evidence in this unique patient population. The use of a predefined treatment protocol in a heart-failure clinic may reduce variability in treatment practices, which may benefit patients and could facilitate future evaluations whether treatment-effects are present (or absent) for certain subgroups of patients [5,20].

### 4.2 Outcome

Prior studies already indicated that a HF diagnosis in a patient with CHD directly relates to an increased risk of death, HTX or VAD implant, and is therefore a valuable prognostic marker in addition to the ACHD anatomic and physiological (AP) classification [6,27,28]. Early and late mortality risk for ACHD-HF patients is even higher when compared to HF patients without CHD. Importantly, ACHD-HF patients utilize more healthcare resources, such as higher emergency department admissions, longer hospital stays, and greater hospital costs [28,29]. Finally, whilst studies indicate that AHCD-HF hospitalizations have increased by 46 % versus 6 % over a 10-year period (due to aging population, disease progression, improved survival amongst others), the use of advanced HF therapies (VAD and/or HTX) decreased in comparison with HF-non-ACHD [30].

This study provides granular event rates for different clinical outcomes across different pre-specified ACHD subgroups over a period of 2.5 years. Remarkably, despite a relatively young age, event rates were high (around 10 % per year for the combined endpoint all-cause mortality, HTX or VAD implant as well as for the occurrence of a HF hospitalization and up to 18 % per year if NYHA progression was also considered).

There were some differences between subgroups, the worst event rates in cyanotic patients and lower mortality rates (albeit with a higher incidence of HTX and VAD) in patients with a systemic right ventricle. These data suggest a distinct clinical trajectory for different subgroups, with transplant or device therapy more readily pursued in patients with a systemic RV [5,31,32], and transplant as an option in patients with a Fontan circulation [33].

Importantly, survival analysis further underscored the prognostic value of HF-related events. HF hospitalization is strongly associated with ‘hard’ adverse clinical endpoints [28,34]. Similarly, persistent NYHA progression was significantly associated with the combined endpoint of all-cause mortality, HTX and VAD implantation and can be considered as an endpoint in a clinical trial. This adds to earlier evidence showing the prognostic value of (baseline) NYHA in ACHD [5,35].

Whether patient-reported outcomes have a place could not be determined from our dataset [36]. Importantly, a sustained increase in diuretic therapy was not related to ‘hard’ endpoints, and should not be used as a surrogate endpoint in clinical trials. However, as other HF medications were escalated concurrently in different patients, this could be a confounding factor, making escalation of diuretic therapy a less ideal parameter of clinical worsening. The incidence of clinical worsening observed in this study can be used for sample size calculations in future clinical studies.

### 4.3 Strengths and limitations

This study captures up-to date medical therapy and clinical outcomes in a real-world setting, offering valuable insight into current treatment practices in ACHD-HF, an area where evidence is still limited. Patients were stratified according to anatomical and pathophysiological subgroups enabling nuanced analysis of how heart failure outcomes differ across ACHD subtypes.

Several limitations are inherent to the retrospective design of this observational study with a relatively small sample size. First, the small sample size and study population from one country reduces statistical power, making it harder to detect significant associations or generalize findings to a larger population. Second, retrospective designs rely on pre-existing data, which may be inconsistent or subject to recall and documentation biases. Third, confounding variables were not adequately controlled, leading to potential misinterpretation of documented associations. Fourth, although we assessed associations between parameters reflecting clinical worsening and traditional endpoints for the overall cohort, larger datasets are required to perform similar analyses for each subgroup. Fifth, the retrospective, observational nature of the study does not allow to draw any conclusions on the prognostic impact of therapeutic interventions.

## 5 Conclusions

This observational study provides insight into current medical therapy in a contemporary cohort of ACHD-HF patients. Beyond death, HTX and VAD implantation, NYHA progression and HF hospitalization emerge as possible valuable endpoints for defining clinical worsening in ACHD patients, whereas diuretics escalation does not. Future studies are required to validate these endpoints for use in randomized controlled clinical trials evaluating medical therapy in this population.

## CRediT authorship contribution statement

**Thibault Bourgeois:** Writing – original draft, Data curation, Conceptualization. **Juliette Hubert:** Data curation. **Pieter De Meester:** Writing – review & editing. **Thibault Petit:** Writing – review & editing. **Els Troost:** Writing – review & editing. **Lucas Van Aelst:** Writing – review & editing. **Filip Rega:** Writing – review & editing. **Philip Moons:** Writing – review & editing. **Werner Budts:** Writing – review & editing. **Alexander Van De Bruaene:** Writing – review & editing, Supervision, Formal analysis, Conceptualization.

## Declaration of competing interest

The authors declare that they have no known competing financial interests or personal relationships that could have appeared to influence the work reported in this paper other t
